# Protective Effect of Hydroxysafflor Yellow A on Nephropathy by Attenuating Oxidative Stress and Inhibiting Apoptosis in Induced Type 2 Diabetes in Rat

**DOI:** 10.1155/2020/7805393

**Published:** 2020-03-11

**Authors:** Maosheng Lee, Hengxia Zhao, Xuemei Liu, Deliang Liu, Jianping Chen, Zengying Li, Shufang Chu, Xinhui Kou, Si Liao, Yuxiu Deng, Huilin Li, Weidong Xie

**Affiliations:** ^1^The Fourth Clinical Medical College, Guangzhou University of Chinese Medicine, Guangzhou 510006, China; ^2^Department of Endocrinology, Shenzhen Traditional Chinese Medicine Hospital, Shenzhen 518033, China; ^3^Shenzhen Key Laboratory of Hospital Chinese Medicine Preparation, Shenzhen Traditional Chinese Medicine Hospital, Shenzhen 518033, China; ^4^Key Lab in Health Science and Technology, Division of Life Science and Health, Tsinghua Shenzhen International Graduate School, Tsinghua University, Shenzhen 518055, China

## Abstract

Diabetic nephropathy (DN) is a serious complication of diabetes mellitus, and its prevalence has been increasing all over the world, which is also the leading cause of end-stage renal failure. Hydroxysafflor yellow A (HSYA) is the main active chemical component of Carthamus tinctorius L., and it is commonly used in patients with cardiovascular and cerebrovascular diseases in China. The aim of this study was to investigate the renal protective effects and molecular mechanisms of HSYA on high-fat diet (HFD) and streptozotocin- (STZ-) induced DN in rats. The DN rats were treated with HSYA for eight weeks. We assessed creatinine (CR), urea nitrogen (UN), glomerular volume, podocyte number, renal inflammation, oxidative stress, and cells apoptosis markers after HSYA treatment. The number of apoptotic cells was measured by the TUNEL assay, and apoptosis-related proteins BAX, caspase-3, and BCL-2 in the renal tissue were analyzed by western blot. The treatment with HSYA significantly decreased fasting blood glucose, CR, UN, and blood lipid profile, including triglyceride and total and low-density lipoprotein cholesterol, even though it did not change the rats' body weights. The western blot results indicated that HSYA reversed the upregulation of BAX and caspase-3 and significantly increased BCL-2 in renal tissue. Moreover, the levels of TNF-*α* and the inflammatory products, including free fatty acids (FFA) and lactic dehydrogenase (LDH) in the HSYA group, were significantly decreased. For the oxidative stress marker, the superoxide dismutase (SOD) markedly increased in the HSYA treatment group, while the malondialdehyde (MDA) in the serum and kidney tissue evidently decreased. In conclusion, HSYA treatment preserved kidney function in diabetic nephropathy in the HFD- and STZ-induced rats. The potential mechanism of renal protective effect of HSYA might be through inhibiting oxidative stress, reducing inflammatory reaction, and attenuating renal cell apoptosis. Our studies present a promising use for Hydroxysafflor yellow A in the treatment of type 2 diabetes mellitus.

## 1. Introduction

Diabetes mellitus (DM), a noncommunicable chronic disease, is a metabolic disease characterized by insulin resistance and *β*-cell dysfunction [1], associated with inflammation caused by overnutrition or other environmental factors [2, 3]. DM would damage blood vessels in the kidneys, leading to kidney damage as well as a decrease in kidney function. Diabetic nephropathy (DN) affects 20-40% of people with DM at any point in time [4]. Type 2 diabetes mellitus (T2DM) accounts for more than 90% of diabetes patients, and nephropathy affects 20% of patients with type 2 diabetes [5].

Actually, numerous factors are involved in the pathophysiology of DN, including hemodynamic alterations, oxidative stress, and inflammation, which might be caused by hyperglycemia and hyperlipoidemia. Chronic hyperglycemia increases oxidative stress and the reaction of glycoxidation and peroxidation. Overproduction of intracellular reactive oxygen species would contribute to macrovascular and microvascular complications of DN [6]. On the other hand, an increase in responsive oxygen species can increase the production of inflammatory cytokines. Previous studies [7, 8] have shown that renal inflammation is critical in promoting the occurrence and development of DN. Moreover, hyperglycemia-induced reactive oxygen species (ROS) can cause apoptosis of podocytes of DN [9], and apoptosis is a potential mechanism of glomerular and tubular cell loss in T2DM patients [10]. Therefore, the medicine with anti-inflammatory and antioxidation effects might significantly improve DN.

**Figure 1:**
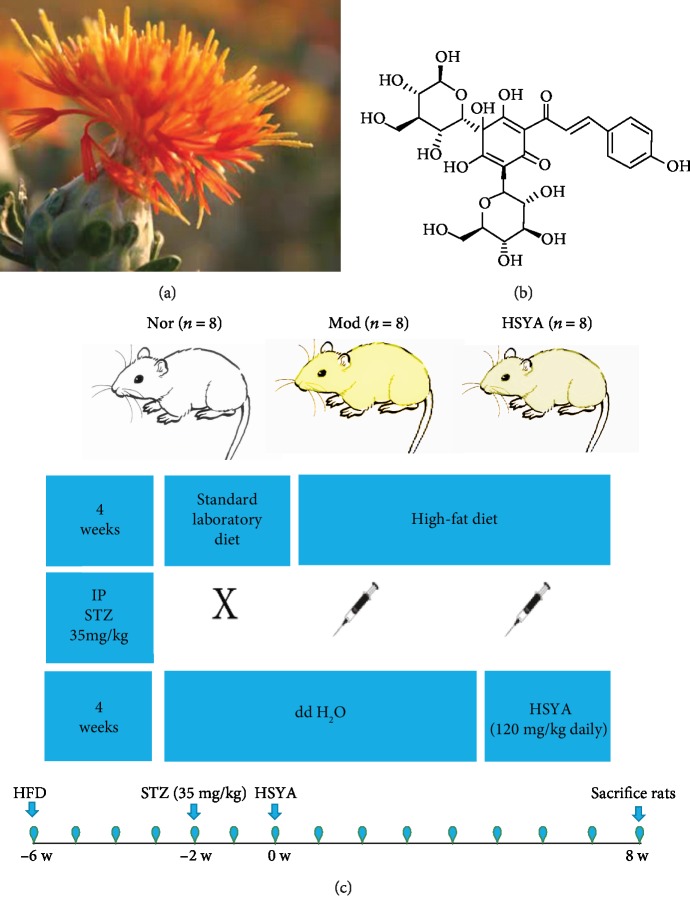
Experimental drug and experimental scheme. (a) The picture of Carthamus tinctorius L. (safflower). (b) The chemical structure (C27H32O16) of Hydroxysafflor yellow A (HSYA). (c) Design of our experimental groups, number of rats, modeling method and time point, the treatment way, and duration. The Nor group rats fed with standard laboratory diet, and the other groups fed with high-fat diet (HFD) in the whole course of the experiment. Nor: normal group; Mod: model group; HSYA group: Hydroxysafflor yellow A group; IP-STZ: intraperitoneal injection of streptozotocin.

Carthamus tinctorius L. (Figure 1(a)) is commonly known as safflower, which is widely used in Traditional Chinese Medicine (TCM) with its effects of anti-inflammatory and antioxidant. Hydroxysafflor yellow A (HSYA), a water-soluble monomer, can be extracted from Carthamus tinctorius L. (molecular formula, C_27_H_32_O_16_; molecular weight, 612.5 g/mol) [11], shown in Figure 1(b). HSYA is the main active chemical component of Carthamus tinctorius L. and is also commonly used in patients with cardiovascular and cerebrovascular diseases in China with its effect of oxygen-free radical scavenging, anti-inflammatory, and antiapoptotic activities [12–14]. HSYA was also selected as an active marker component for controlling the quality of safflower in Chinese Pharmacopoeia (The State Pharmacopoeia Commission of China, 2015).

HSYA was widely distributed in many vital organs. It is evidenced that HSYA has many effects for protecting the organs, such as protecting the myocardial injury [15], cerebral ischemia-reperfusion injury [16], and alcohol-induced liver injury [17] by inhibiting oxidative stress, inflammatory reaction, and apoptosis. Additionally, the oxidative stress and inflammation hugely contributed to T2DM; therefore, HSYA might be a potential antidiabetic drug.

However, there is still no study of HSYA for the treatment of T2DM. Furthermore, HSYA played a key role in protecting acute kidney injury [18]. However, there is a lack of studies about T2DM-induced chronic kidney disease and the treatment of HSYA. Thus, we investigated the effect of HSYA on T2DM and DN in rats and further explored its mechanism.

In this study, we performed rats' modeling of high-fat diet- (HFD-) and streptozocin- (STZ-) induced nephrotoxicity and how to ameliorate the DN effect by using HSYA. After 8 weeks of treatment, we found that HSYA can significantly reduce fasting blood glucose and improve oxidative stress and inflammatory state, which might be the potential mechanism of HSYA protecting against DN.

## 2. Material and Methods

### 2.1. Animal Experiment

Twenty-four adult Wistar rats (weighted, 150 ± 20 g) were purchased from Guangdong Medical Laboratory Animal Center (Guangzhou, China). All rats were kept in a specific-pathogen-free (SPF) animal laboratory with free access to food and water.

Rats were divided equally into three groups, and they were treated in different methods as depicted in Figure 1(c). The HFD (60% fat) was purchased from Guangdong Medical Laboratory Animal Center (Code D12492, Guangzhou, China). STZ was obtained from Sigma (Sigma Chemical Co., St., USA) and HSYA was obtained from Nanjing Daofu Biotechnology Co., Ltd. (CAS 78281-02-4, purity ≥ 98%, HPLC, Nanjing, China).

After modeling, the T2DM rats were daily orally administrated with HSYA (120 mg/kg) or normal saline for 8 weeks. After eight weeks of treatment, all rats were anaesthetized by sodium pentobarbital and blood were sampled from the aorta abdominalis. Kidney tissues were dissected, some of them will be frozen at -80°C immediately, and the others were immersed in 10% neutral-buffered formalin for further studies.

All animal procedures were conducted with protocol approval from the Institutional Animal Care and Use Committee (IACUC) of Tsinghua University, according to the National Institute of Health ethical guidelines, and all efforts were made to minimize animal suffering.

### 2.2. Biomedical Analysis

Fasting blood glucose (FBG) was measured by a glucometer (Roche Diagnostic Products Co. Ltd, Shanghai, China), and blood samples were collected from the tail vein of the 12 h fasted rats. The creatinine (CR), urea nitrogen (UN), triglycerides (TG), total serum cholesterol (TC), low-density lipoprotein cholesterol (LDL-C), and lactate dehydrogenase (LDH) in the serum were evaluated by rat enzyme-linked immune sorbent assay (ELISA) kit according to the manufacturer's instructions (BioSino Bio-Technology & Science Inc. Beijing, China; Beijing Solarbio Science & Technology Co., Ltd. Beijing, China). The TNF-*α*, free fatty acids (FFA), and LDH were detected by rat ELISA kit according to the manufacturer's instructions (Wuhan Mershack Biotechnology Co., Ltd., Wuhan, China; Beijing Solarbio Science & Technology Co., Ltd., Beijing, China). The superoxide dismutase (SOD) and malondialdehyde (MDA) in the serum and kidney were determined by the WST-8 method. The renal glycogen (RG) in the kidney were detected by anthrone method according to the manufacturer's instructions (Solarbio Science & Technology Co., Ltd., Beijing, China).

### 2.3. Histological Analysis and Determination of Apoptosis

The kidney tissues, after 48 hours fixation, were prepared into paraffin sections of 4.5 *μ*m, stained with hematoxylin-eosin (HE) (Sigma-Aldrich), and assessed on a fluorescence microscope (Nikon Eclipse E100 microscope; Nikon, Tokyo, Japan).

Kidney cell apoptosis was detected by terminal deoxynucleotidyl transferase-mediated dUTP nick-end labeling (TUNEL) assay using the tissue paraffin sections. Kidney slides, made into 3.5 *μ*m paraffin sections after dehydration, were incubated with the TUNEL reaction mixture in a humidified chamber for 60 minutes at 37°C in light avoidance condition. Renal sections were rinsed in phosphate-buffered saline (PBS), and the nuclei were stained with DAPI at a concentration of 460 nm. Apoptosis was quantified by calculating the percentage of TUNEL-positive nuclei in an average of 10 high-power fields for each section in random.

### 2.4. Western Blot Analysis

The expression of kidney proteins, including BAX (20 kDa, CST, USA), caspase-3 (19 kDa, CST, USA), and BCL-2 (26 kDa, CST, USA), was analyzed by western blotting. Briefly, 100 mg of kidney tissues was homogenized in RIPA buffer for 10 minutes followed by centrifugation at 12000 rpm for 10 minutes at 4°C. The total protein was extracted from the kidney, and the concentrations were detected by using a Bradford Protein Assay Kit (Beyotime Biotechnology, Shanghai, China). Then, the goal proteins were separated on 10% SDS-PAGE gels and then transferred onto nitrocellulose membranes. The membranes were incubated in blocking solution containing 5% nonfat dried milk for 1.5 h at room temperature, and the following primary antibodies were used for overnight incubation at 4°C. Antibodies against BAX (#2774), caspase-3 (#9662), and BCL-2 (#3498) were obtained from Cell Signaling Technology and used at a dilution of 1 : 1000; antibody against GAPDH was used at a dilution of 1 : 1000. Then, the membranes were washed in TBST and incubated with appropriate horseradish peroxidase-conjugated secondary antibody. After incubation with the secondary antibody for 1.5 hours at room temperature, the protein bands were exposed to the chemiluminescent reagent (ECL) for about 5 minutes. Protein expression was measured with fluorescence captured on X-ray photographic film in a dark room. The band densities were quantified, and a densitometry analysis was performed by using ImageJ software (NIH, Bethesda, MD, United States).

### 2.5. Statistical Analysis

SPSS (Statistical Product and Service Solutions) statistics 22.0 and GraphPad Prism 8.0 software (San Diego, CA, United States) were applied for the data statistical analysis and graphics. All parameters were expressed as mean ± SD in each group. Unpaired *t*-test was used to analyze statistical comparisons between two groups when necessary. Multiple comparisons were compared by one-way (or two-way) analysis of variance (ANOVA) followed by Bonferroni's hoc tests. Statistically significant changes were classified as significant (^∗^) when *P* < 0.05.

## 3. Results

### 3.1. Antihyperglycemic Effect of HSYA in T2DM Rats

**Figure 2:**
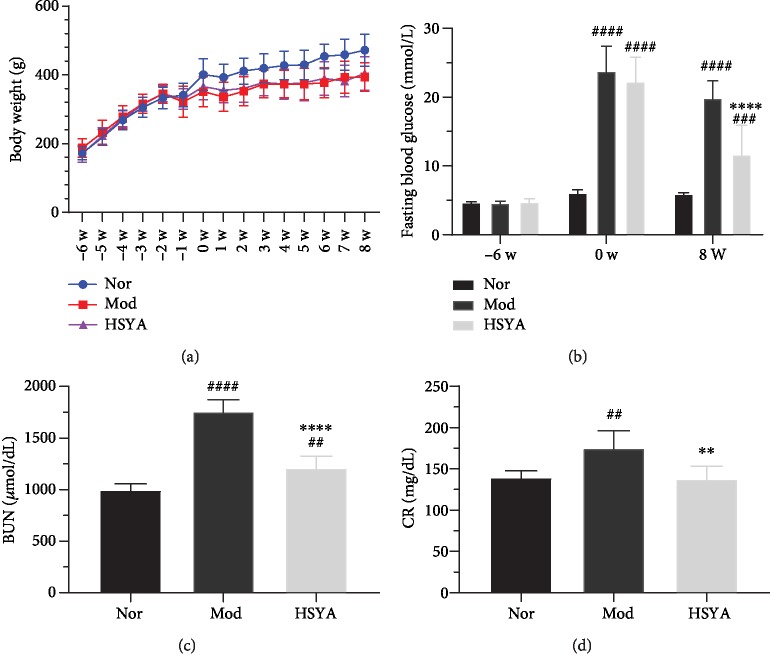
Effect of HSYA on T2DM and diabetic nephropathy (DN) rats. (a) Body weight of the three groups. (b) Fasting blood glucose level in three time points. ^####^*P* < 0.0001*vs.* Nor; ^∗∗∗∗^*P* < 0.0001*vs.* Mod (HSYA 120 mg/kg, intragastric administration) (*n* = 8 rats/group). (c, d) Blood urea nitrogen (BUN) and serum creatinine (CR) were detected in each group after 8 weeks of treatment. ^##^*P* < 0.01 and ^####^*P* < 0.0001*vs.* Nor; ^∗∗^*P* < 0.01 and ^∗∗∗∗^*P* < 0.0001*vs.* Mod. Results are presented as means ± SD and *n* = 8 in each group.

The rats' body weight was conducted in all courses, the normal (Nor) group rats were significantly higher than the T2DM groups after STZ modeling although there was no significant statistical difference between the model (Mod) and HSYA group (*P* > 0.05). We monitored the FBG level to assess the effects of HSYA on T2DM rats. After 6 weeks T2DM modeling (-6w to 0w), the FBG of modeling rats conformed to the T2DM model standard, and the FBG of all the diabetic rats was more than 16.8 mmol/L (Figure 2(b)). At the end of the experiment, the HSYA group showed a 46.34% (*P* < 0.0001) decrease in FBG level when compared to the Mod group (15.12%).

### 3.2. HSYA Improves Renal Function in DN Rats

As the results shown in Figures 2(c) and 2(d), the levels of blood urea nitrogen (BUN) and serum creatinine (CR) were significantly increased in the HSYA group. Compared to the Nor group, the Mod group developed drastic renal dysfunction as indicated by increased BUN (1757 ± 113.9 *μ*mol/dL) and CR (175.1 ± 21.35 *μ*mol/L) levels. After the HSYA treatment, both the BUN (1209 ± 117.3 *μ*mol/dL) and CR (137.8 ± 15.54 *μ*mol/L) levels were significantly decreased, which indicated that HSYA can ameliorate the DN in a certain extent.

### 3.3. HSYA Protect against the Glomerular Structure in DN Rats

**Figure 3:**
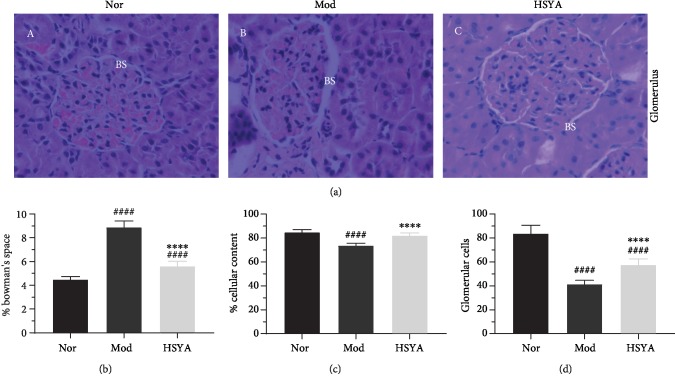
HSYA-protective effects against diabetic nephropathy. (a) Paraffin tissue sections stained against H&E (×400, *n* = 6 each group). (b) Dilated Bowman's space (BS) in diabetic glomeruli comparable to normal group. Image size measurements (ImageJ software) of Bowman's space and cellular contents in relation to the whole glomerulus size. % Bowman's space was remarkably increased in the Mod group and decreased gradually after HSYA treatment. (c) In the diabetic glomerulus, % size of cellular content was markedly decreased and then increased observably with HSYA administration. (d) The glomerular number increased after HSYA treatment. ^####^*P* < 0.0001*vs.* Nor; ^∗∗∗∗^*P* < 0.0001*vs.* Mod. Results are presented as means ± SD and *n* = 6 in each group.

In order to explore the results of the T2DM rats glomerular structure, we stained the kidney paraffin sections with H&E. Expectantly, the structural changes in the glomeruli emerged in T2DM rats when compared with the Nor group rats. Glomerular cellular contents and Bowman's space size associate directly with the glomerular good functional state. As shown in Figure 3(a), our results revealed a palpable dilatation of Bowman's space (BS) in T2DM rats when compared with the Nor group rats (Figure 3(b)). At the same time, the glomerular cellular contents in T2DM rats were remarkably decreased. The ImageJ software was used for a quantitation of % Bowman's space, % cellular contents, and podocyte number in glomeruli. The % Bowman's space and % cellular contents in T2DM glomeruli markedly increased (8.89% ± 0.53%) compared to the Nor group (4.51% ± 0.22%, *P* < 0.0001), and it was recovered in a certain extent after HSYA treatment (5.62% ± 0.40%, *P* < 0.0001) (Figures 3(b) and 3(c)). Additionally, the podocyte number in T2DM glomeruli showed an obviously abatement (59.60%, rep.) when compared to the Nor group rats; however, it was miraculously recovered to normalization after HSYA treatment (Figure 3(d)) (*vs.* Mod *P* < 0.0001).

### 3.4. HSYA Reduces Apoptosis in DN Rats

**Figure 4:**
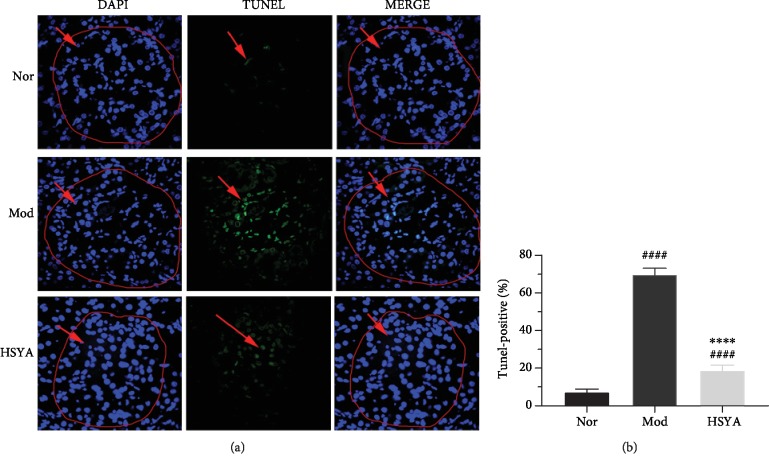
HSYA ameliorates glomerular apoptosis induced by DN. Representative images of the TUNEL staining in the normal (Nor), model (Mod), and Hydroxysafflor yellow A (HSYA) groups. (a) (first column) DAPI staining (*blue*) indicates total nuclei, (*middle column*) apoptotic nuclei detected by TUNEL staining (*green*), and (*last column*) overlay of both types of staining. (b) The number of TUNEL-positive myocytes was performed as a percentage of total nuclei detected by DAPI staining (fluorescence microscopy, magnification ×60). ^####^*P* < 0.0001*vs.* Nor; ^∗∗∗∗^*P* < 0.0001*vs.* Mod. Results are presented as means ± SD and *n* = 6 in each group.

To observe the apoptosis of glomerular cells in T2DM rats, TUNEL staining was conducted in our study. Staining of kidney sections was performed to visualize the DNA fragmentation in situ. In the Mod group, the TUNEL assay depicted a marked increase in TUNEL-positive cells in T2DM rats' kidney when compared with the Nor group (*P* < 0.0001); expectingly, they were remarkably reduced in HSYA group after HSYA treatment (Figures 4(a) and 4(b)) (*vs.* Mod *P* < 0.0001, from 69.93 ± 3.22 to 18.92 ± 2.67). The HSYA-treated group exhibited fewer numbers of TUNEL-positive cells.

### 3.5. HSYA Inhibits Apoptosis and TNF-*α* in DN Rats

**Figure 5:**
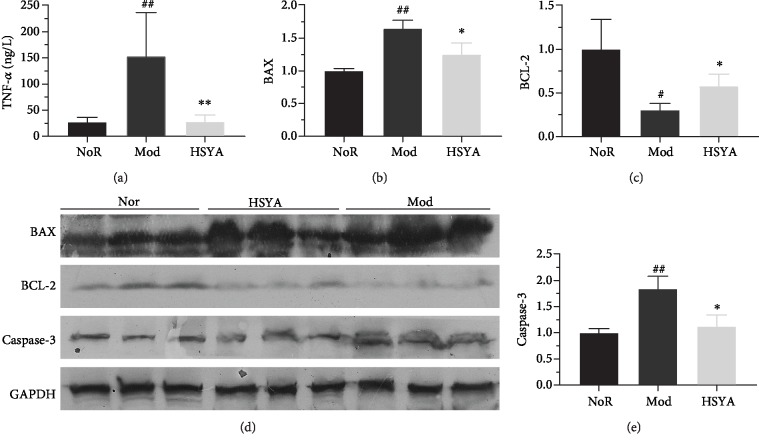
Effects of HSYA on TNF-*α*, BAX, BCL-2, and casepase-3 expression in renal tissue. (a) Tumor necrosis factor (TNF-*α*) level was significantly increased in the Mod group and decreased markedly after using HSYA. (b–e) Representative immunoblots of BAX, BCL-2, casepase-3, and GAPDH in the renal tissue, respectively. Data are described as mean ± SD (*n* = 3). (b) Relative expressions of BAX in renal tissue. (c) Relative expressions of BCL-2 in renal tissue. (d) Relative expressions of casepase-3 in renal tissue. All data are described as mean ± SD (*n* = 3). ^#^*P* < 0.05 and ^##^*P* < 0.01*vs.* Nor; ^∗^*P* < 0.05 and ^∗∗^*P* < 0.01*vs.* Mod.

Figure 5(a) shows the TNF-*α* overexpression in the T2DM rats' kidney when compared with the Nor group rats (*P* < 0.01), while HSYA could suppress the TNF-*α* level in T2DM rats (*vs.* Mod *P* < 0.01). In addition, western blot analysis showed that the BAX and caspase-3 expression significantly increased in the kidney of T2DM rats compared to the normal rats (*P* < 0.01); however, they were both downregulated after eight weeks of HSYA treatment (Figures 5(b)–5(d)) (*vs.* Mod *P* < 0.05). Moreover, BCL-2, the inhibitor of apoptosis, was restrained in the kidney of T2DM rats, and HSYA could upregulate BCL-2 in a certain extent (Figure 5(d) and 5(e)) (*vs.* Mod *P* < 0.05).

### 3.6. HSYA Ameliorate Hyperlipemia and Inflammatory Reaction in DN Rats

**Figure 6:**
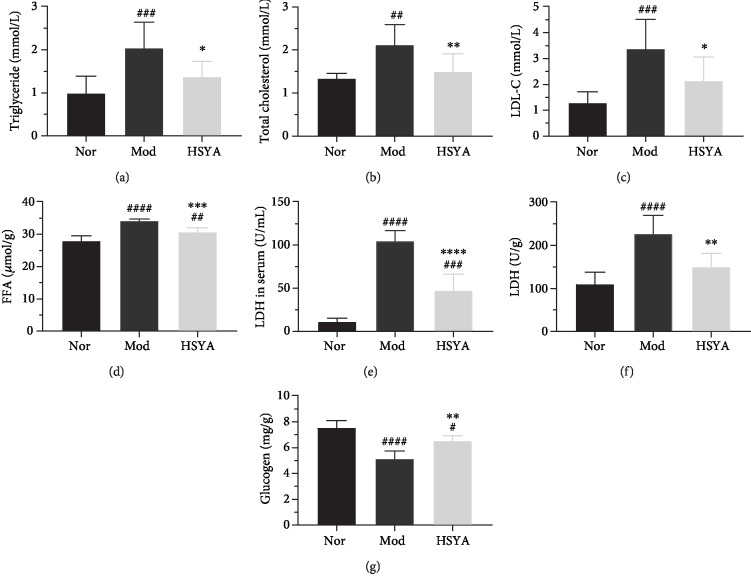
Effects of HSYA on the blood lipid profile and some critical inflammatory products. (a) Triglycerides (TG) level. (b) Total cholesterol (TC) level. (c) Low-density lipoprotein cholesterol (LDL-C) level. (d) Free fatty acid (FFA) level in renal tissue. (e, f) Lactate dehydrogenase (LDH) level in serum and renal tissue. (g) The glucogen level in renal tissue. The total TC, TG, LDL-C, and LDH levels in DN rat's blood serum were tested, and the FFA, LDH, and glucogen in the renal tissue were measured in each group after 8 weeks of treatment. Results are presented as means ± SD and *n* = 8 (*n* = 6 in renal tissue) in each group. ^#^*P* < 0.05, ^##^*P* < 0.01, ^###^*P* < 0.001, and ^####^*P* < 0.0001*vs.* Nor; ^∗^*P* < 0.05, ^∗∗^*P* < 0.01, ^∗∗∗^*P* < 0.001, and ^∗∗∗∗^*P* < 0.0001*vs.* Mod.

In order to estimate the effects on hyperlipemia and inflammatory reaction, the blood lipid profile and some major inflammatory products in kidney tissue were detected. In the Mod group, the blood lipid profile, including TG, TC, and LDL-C, was significantly increased compared with the Nor group (Figures 6(a)–6(c)) (*P* < 0.01 or *P* < 0.001), while HSYA has a good effect of reducing blood lipid profile (*vs.* Mod, *P* < 0.05 or *P* < 0.01).

Some major inflammatory products in the T2DM kidney, such as LDH, FFA, and glycogen, had a significant change when compared with the Nor group (*P* < 0.0001). HSYA treatment could reduce the cumulation of FFA and LDH and the consumption of glycogen in T2DM rats (Figures 6(d), 6(f), and 6(g)) (*vs.* Mod, *P* < 0.01 or *P* < 0.001). Furthermore, HSYA could significantly reduce the LDH level in the serum for the sake of reducing damage to target organs such as the kidney (Figure 6(e)) (*vs*. Mod, *P* < 0.0001). Taken together, HSYA could ameliorate hyperlipemia and suppresses the inflammatory response by reducing the accumulation of inflammatory product.

### 3.7. HSYA Inhibits Reactive Oxygen Species (ROS) in DN Rats

**Figure 7:**
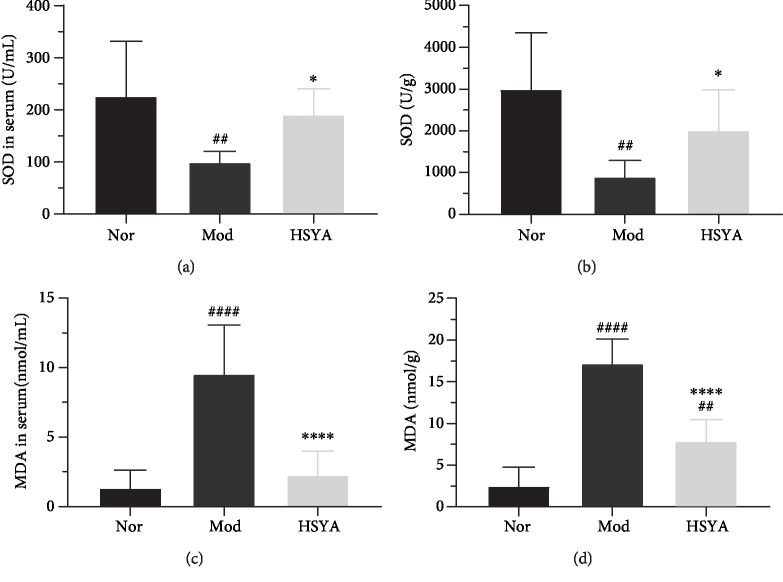
HSYA inhibits reactive oxygen species (ROS) in DN rats. (a, b) The activity of superoxide dismutase (SOD) in serum and renal tissue. (c, d) The malondialdehyde (MDA) levels in serum and kidney tissue were determined by the WST-8 method. Results are presented as means ± SD and *n* = 8 (*n* = 6 in renal tissue) in each group. ^#^*P* < 0.05, ^##^*P* < 0.01, and ^####^*P* < 0.0001*vs.* Nor; ^∗^*P* < 0.05, ^∗∗^*P* < 0.01, and ^∗∗∗^*P* < 0.0001*vs.* Mod*..*

ROS production contributes to DN development; to further explore the effect of HSYA on ROS, the serums MDA and SOD, as well as the MDA and SOD levels in kidney tissue, were detected. Compared with the Nor group rats, the serum MDA and kidney tissue's MDA levels were upregulated, and the serum SOD and kidney tissue's SOD levels were downregulated expectantly (Figures 7(a)–7(d)) (Mod *vs.* Nor, *P* < 0.01 or *P* < 0.0001). The HSYA treatment can reverse the upregulation of MDA and the downregulation of SOD (HSYA *vs.* Mod, *P* < 0.05 or *P* < 0.0001).

## 4. Discussion

It is known to all that DN is mainly characterized by oxidative stress, inflammation, and apoptosis that eventually results in glucose toxicity, lipid toxicity, and their related complications. In our current study, it is the first time to be evidenced that HSYA has a certain degree of antidiabetic effect, and nephrotoxicity induced by T2DM could be rescued by the HSYA treatment.

The HFD- and STZ-induced T2DM rats were commonly used models to study obesity-related T2DM [19]. In our study, the diabetic rats exhibited significantly higher FBG levels (Figure 1(b)), higher blood lipid profile levels (Figures 6(a)–6(c)), and severe kidney impairment (Figures 2(c), 2(d), and 4(a)) compared to the normal rats. As the previous studies [20–22] described, T2DM-induced nephrotoxicity in rats was characterized by high levels of urea, creatinine, lipid profile, and glucose in serum. All these suggest that the model for DN (related to T2DM) was created successfully.

Glomerular cell loss is deemed to be an outcome of T2DM-induced nephrotoxicity [23, 24], and the vasodilatation of the glomerulus could induce a podocyte mechanical tractive leading to foot process effacement and cellular detachment. The previous studies [25–27] suggest that podocyte tractive brings a decreased podocyte nephrin expression, which will affect the slit diaphragm protein and obstruct the function of glomerular filtration, resulting in proteinuria. Bowman's space would be enlarged by the accumulation of edematous fluids in the period of diabetes. The increase of urine in diabetic rats exceeded the normal load of renal reabsorption, resulting in the accumulation of renal edema fluid. HSYA treatment could alleviate renal injury by decreasing CR and BUN levels to protect against the glomerular cell loss.

BCL-2 was one main of the antiapoptotic members, and BAX was one major of the proapoptotic members; the balance between them would decide the induction of the intrinsic pathways of apoptosis [28]. Caspase's family are crucial mediators of programmed apoptosis. Among them, caspase-3 is a frequently activated death protease, catalyzing the specific cleavage of numerous pivotal cellular proteins [29]. Our results showed that HSYA treatment can decrease the expression of BAX and increase the gene expression of BCL-2. Naturally, the BCL-2/BAX ratio was lower in kidney in HSYA group rats. Furthermore, HSYA markedly decreased the expression of caspase-3, which in turn inhibited the apoptosis of cells, as the TUNEL staining depicted.

It has been evidenced [30, 31] that HSYA could be protected against kidney injury by its anti-inflammatory action. As an inflammatory mediator, TNF-*α* has a high correction with the extensive tubular damage in kidney [32, 33]; as the previous study suggested, HSYA could inhibit the expression of tumor necrosis factor alpha (TNF-*α*) [34]. Expectantly, HSYA distinctly reduced the TNF-*α* expression in the T2DM rats in our study. When the T2DM kidney sustained in a continuous inflammatory state, it will lead to a large number of inflammatory products' accumulation in the organ, further accelerating the inflammatory process and cell apoptosis. In our study, the inflammatory products, such as the LDH and FFA, were accumulated in the kidney of T2DM rats, which accelerated the oxidative hydrolysis of sugar and fat, and glycogen in kidney tissue is consumed (Figures 6(d)–6(g)). Similarly, the hypercholesterolemia and hypertriglyceridemia may also play a pathogenic role in progressive glomerular damage, and reduction in TG and TC can reduce the rate of decline of glomerular filtration rate (GFR) in patients with diabetic nephropathy. Lipid-lowering therapy in rat models of hypertension can reduce urinary albumin excretion rate (UAER) [35]. The statin (cholesterol-lowering drug) treatment could decrease the UAER and microalbuminuria in DN patients [36]. Furthermore, reduction in hypertriglyceridemia applying fibrates therapy can abate UAER rise in T2DM patients [37]. Expectingly, HSYA can significantly lower the level of blood lipid profile in T2DM rats in our study, which has a similar result with the previous study [38]. Taken together, HSYA could ameliorate hyperlipemia and suppresses the inflammatory response by reducing the accumulation of inflammatory product.

Additionally, consistent with other studies [39–41], we found that the T2DM kidney and serum revealed a significant levels of oxidative stress markers like MDA and SOD. The persistent high-intensity oxidative stress was deemed to be an inducement of inflammatory and apoptotic, which would lead to cell loss [42, 43]. As the previous study depicted, HSYA could significantly alleviate cerebral ischemia in rats by decreasing the level of MDA and increasing the level of SOD [44]. HSYA also can reduce Ca2^+^ overload-induced ROS generation, which could improve mitochondrial energy metabolism and increase ATP level and the respiratory control ratio [45]. In this study, our results suggest that HSYA significantly protected against renal injury from oxidative stress driven by induced-T2DM rats.

On the other hand, in a toxicity test of HSYA, the subchronic toxicity of HSYA with 90 days of repeated intraperitoneal injections in rats showed that there is not an obvious pathological change in liver histological analysis and has no other organ injury at a dose of 180 mg/kg [46]. Evidently, HSYA is a safe drug, both in injection and oral administration, and its second clinical study has been approved by the SFDA for the therapy (for injection) in patients with brain blood vessel disease. HSYA was poorly absorbed with an oral bioavailability value of 1.2% [47]; however, another study suggested that HSYA might be a potential therapeutic drug for obesity, and the gut microbiota may be a potential territory for targeting of HSYA [48]. Furthermore, the half-lives of HSYA are very short and 90% of the HSYA are eliminated within six hours in normal rats; however, HSYA was with high uptake and eliminated slowly in the rats with blood stasis syndrome [49]. Diabetic patients have a certain blood stasis syndrome, and HSYA was primarily absorbed in the small intestine; poor blood circulation will prolong the retention time of HSYA and increased of HSYA's absorption [50]. Therefore, a higher dose of HSYA (120 mg/kg) was used in this study, and it was also confirmed that this dose demonstrated a remarkable antidiabetic effect.

## 5. Conclusions

In conclusion, our results revealed that Hydroxysafflor yellow A could protect against diabetic nephropathy in HFD- and STZ-induced rats. The potential protective mechanism of the kidney may be through inhibiting oxidative stress, inflammatory reaction, and apoptosis. Our studies present a promising use for Hydroxysafflor yellow A in the treatment of type 2 diabetes mellitus.

## Data Availability

The data used to support the findings of this study are available from the corresponding author upon request.

## Abbreviations

STZ: Streptozotocin
DM: Diabetes mellitus
ROS: Reactive oxygen species
HDL: High-density lipoproteins
LDL: Low-density lipoproteins
DN: Diabetic nephropathy
HSYA: Hydroxysafflor yellow A
HFD: High-fat diet
CR: Creatinine
UN: Urea nitrogen
TUNEL: TdT-mediated dUTP nick-end labeling
FFA: Free fatty acids
LDH: Lactic dehydrogenase
SOD: Superoxide dismutase
MDA: Malondialdehyde
DM: Diabetes mellitus
T2DM: Type 2 diabetes mellitus
ROS: Reactive oxygen species
FBG: Fasting blood glucose
TG: Triglycerides
TC: Total serum cholesterol
LDL-C: Low-density lipoprotein cholesterol
ELISA: Enzyme-linked immune sorbent assay
RG: Renal glycogen
HE: Hematoxylin-eosin.

## Supplementary Materials

Supplementary Materials Protective effect of Hydroxysafflor yellow A on nephropathy by attenuating oxidative stress and inhibiting apoptosis in induced type 2 diabetes in rat. Hydroxysafflor yellow A (HSYA) is the main active chemical component of Carthamus tinctorius L. HSYA treatment preserved kidney function in diabetic nephropathy in the HFD- and STZ-induced rats. The potential mechanism of renal protective effect of HSYA might be through inhibiting oxidative stress (upregulated SOD and downregulated MDA), reducing inflammatory reaction (TNF-*α*, FFA, and LDH) and attenuating renal cell apoptosis (upregulated BCL-2 and downregulated BAX and caspase-3).
