# Supplementary figures and images for: Protective Effect of Hydroxysafflor Yellow A on Nephropathy by Attenuating Oxidative Stress and Inhibiting Apoptosis in Induced Type 2 Diabetes in Rat
Source: Oxid Med Cell Longev. 2020 Mar 11;2020:7805393. doi: 10.1155/2020/7805393 (PMC7091558; doi:10.1155/2020/7805393)

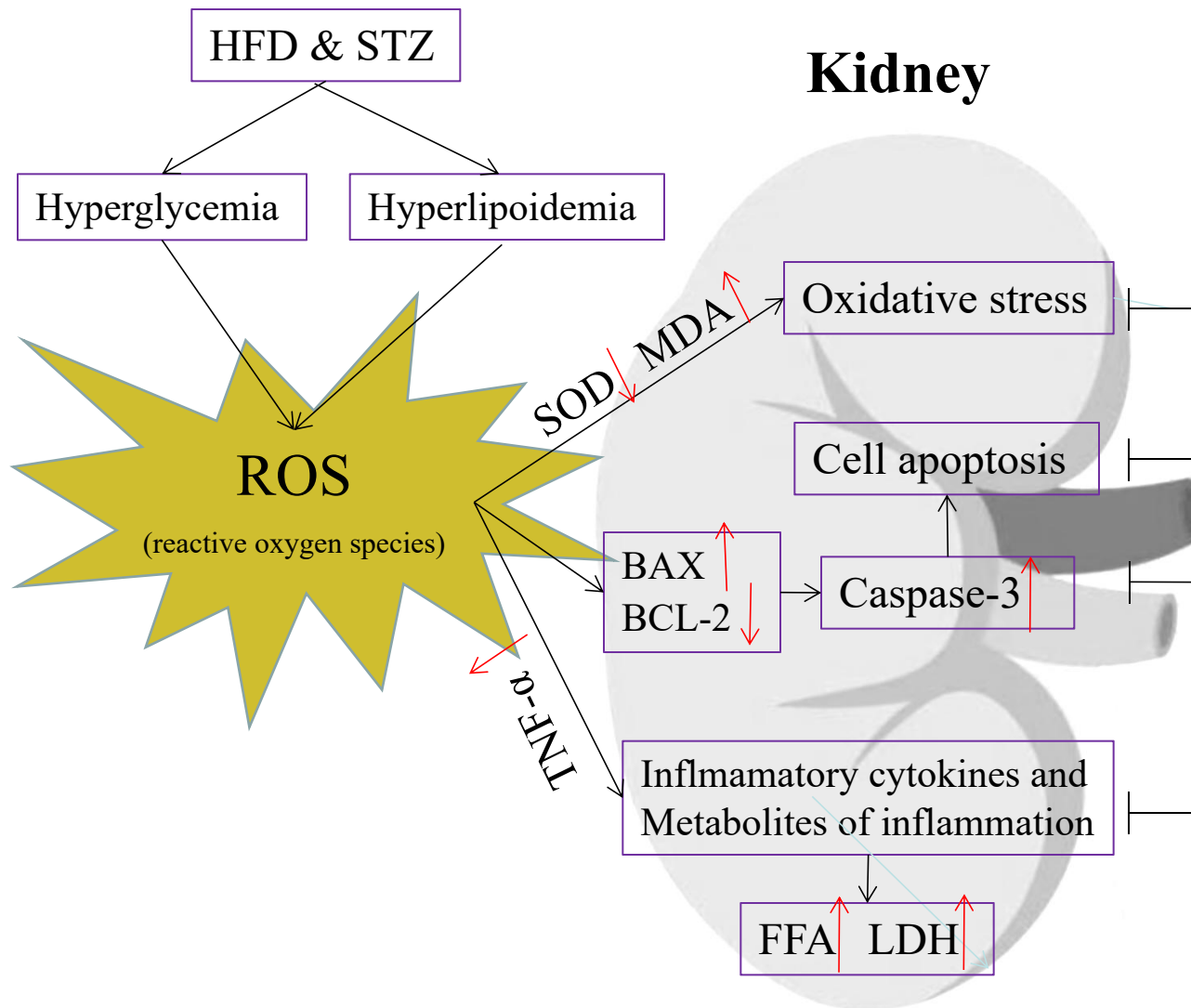

## Kidney

## Carthamus tinctorious L.

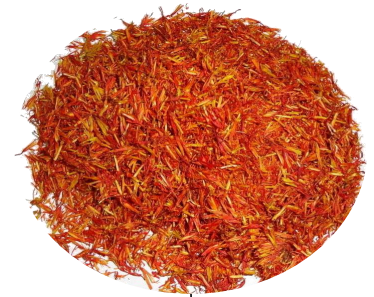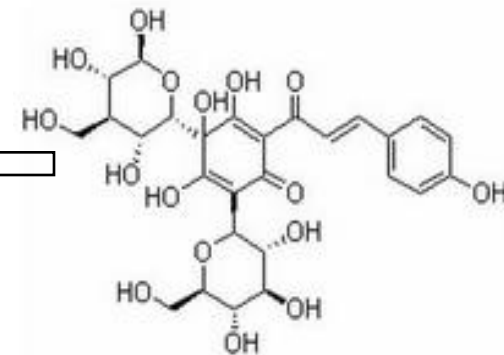

## Hydroxysafflor yellow A

## Diabetic nephropathy

Supplement: Supplementary Materials — Protective effect of Hydroxysafflor yellow A on nephropathy by attenuating oxidative stress and inhibiting apoptosis in induced type 2 diabetes in rat. Hydroxysafflor yellow A (HSYA) is the main active chemical component of Carthamus tinctorius L. HSYA treatment preserved kidney function in diabetic nephropathy in the HFD- and STZ-induced rats. The potential mechanism of renal protective effect of HSYA might be through inhibiting oxidative stress (upregulated SOD and downregulated MDA), reducing inflammatory reaction (TNF-α, FFA, and LDH) and attenuating renal cell apoptosis (upregulated BCL-2 and downregulated BAX and caspase-3). [file 7805393.f1.pdf]
